# Validity and reliability of the Indonesian version of the Scale for the Assessment of Negative Symptoms

**DOI:** 10.3389/fpsyt.2022.1045635

**Published:** 2023-01-20

**Authors:** Mustafa M. Amin, Elmeida Effendy, Vita Camellia, Munawir Saragih, Ahmad Kamil, Rusly Harsono

**Affiliations:** ^1^Department of Psychiatry, Faculty of Medicine, Universitas Sumatera Utara, Medan, Indonesia; ^2^Department of Mechanical Engineering, Faculty of Engineering, Universitas Sumatera Utara, Medan, Indonesia; ^3^Pediatric Critical Care Medicine, School of Medicine, Stanford University, Stanford, CA, United States

**Keywords:** schizophrenia, SANS, validity, reliability, negative symptoms

## Abstract

**Background:**

Negative symptoms have long been conceptualized as a core aspect of schizophrenia. Despite widespread recognition of the status of these symptoms as independent dimensions of schizophrenia, they are sometimes difficult to distinguish from depression or cognitive impairment. Therefore, objective assessment of schizophrenia symptoms is critical by obtaining a valid and reliable Indonesian version of the SANS instrument. This study aimed to determine the content validity, concurrent, internal consistency reliability, inter-rater, cut-off value, sensitivity, and specificity of the SANS instrument.

**Methods:**

This is a diagnostic study using the cross-sectional method to determine the relationship between the SANS and PANSS instruments on the negative symptom subscale. It was located at the Prof. Dr. M. Ildrem Mental Hospital of North Sumatera Province.

**Results:**

Of the 400 subjects, 67.5% were males, and the median age of the subjects was 37 years (18–45). The results of the content validity test were good (mean I-CVI=1.00), and the concurrent validity test comparing the SANS and PANSS instruments on the negative symptom subscale obtained significant results (*p* < 0.001) with a strong correlation (*r* = 0.763). Additionally, the consistency reliability test had a very high internal score (Cronbach alpha = 0.969), the overall inter-rater reliability test was “very good” (ICC = 0.985), and the cut-off value was 10.5 with sensitivity and specificity values of 72.9 and 77.9%, respectively.

**Conclusion:**

The Indonesian version of the SANS instrument is valid and reliable for measuring negative symptoms in people with schizophrenia in Indonesia.

## 1. Introduction

Schizophrenia is a mental disorder characterized by positive symptoms comprising delusions, hallucinations, and disorganized speech, negative symptoms, including flat affect, avolition, speech, and language poverty, social withdrawal, and cognitive deficits covering attention deficit, and impaired executive function (1). In 2013, it was included in the top 25 diseases that are the main causes of disability worldwide. The lifetime prevalence was relatively low (median 4.0 per 1,000 people), and the worldwide prevalence ranges from 2.6 to 6.7 per 1,000 people (2). The basic health data (RISKESDAS) in 2018, conducted by the Ministry of Health of the Republic of Indonesia (KEMENKES RI), found that the prevalence of schizophrenia was 7.0 per 1,000 households. This was significantly increased compared to the prevalence of schizophrenia according to RISKESDAS in 2013, which was 1.7 per 1,000 households. The highest prevalence of RISKESDAS 2018 was in Bali and Yogyakarta at 11.0 and 10.7 per 1,000 households, respectively (3).

Negative symptoms have long been conceptualized as a core aspect of schizophrenia. Despite widespread recognition of these symptoms as an independent dimension of schizophrenia, they are sometimes difficult to distinguish from depression or cognitive impairment. Additionally, the pathogenetic mechanisms remain unknown, available treatments' effectiveness was far from satisfactory, and these symptoms were considered difficult to assess reliably. Furthermore, the main function of negative symptoms in the patient's functional outcome was often overlooked or unknown (4–6).

Several scales and instruments have been proposed and developed to facilitate screening for schizophrenia. These instruments reflect different understandings of how the complication can be well defined and classified according to its symptoms. The scale developed for schizophrenia mainly focuses on assessing a patient through positive and negative symptoms. The *Positive and Negative Syndrome Scale* (PANSS), the *Scale for Assessment of Positive Symptoms* (SAPS), and the *Scale for the Assessment of Negative Symptoms* (SANS) have been developed to assess symptoms of schizophrenia objectively. Because this scale is sensitive to changes in symptoms, it is considered the “gold standard” in studies for the treatment of schizophrenia (4, 6).

Nancy Andreasen developed the SANS in 1982 to assess five common domains of negative symptoms, including alogia, flat affect, avolition-apathy, anhedonia-asociality, and decreased attention. This scale has high interrater reliability globally (0.838) and good overall internal consistency of the items (Cronbach's = 0.885). Therefore, the SANS is a valid and reliable scale to measure the development of predominantly negative symptoms in schizophrenic patients (7).

Many studies have validated SANS into several versions in different countries. Previous studies conducted by Philips et al. validated SANS and measured the validity and reliability of the Chinese version of the SAPS and SANS. In this study, the overall score of the Chinese version of the SANS had high inter-rater reliability (0.93), test-retest reliability (0.88), and good overall internal consistency of the items (Cronbach = 0.96) (8). Meanwhile, the studies conducted by Norman et al., which compared the interrater reliability of SAPS, SANS, and PANSS, found that the global score of SANS interrater reliability was lower than the original journal score (0.68) (9). The study in Thailand conducted by Thammanard Charernboon in 2019 obtained a good overall internal consistency score of items (Cronbach's = 0.95) (10).

SANS is one of the most widely used negative symptom instruments in clinical trials and practice. This instrument was developed specifically to assess negative symptoms in schizophrenia and has been translated into many languages. However, a study on the validity and reliability of the Indonesian version of the SANS has never been conducted. Therefore, it is necessary to obtain a valid and reliable version of the Indonesian SANS instrument.

## 2. Methods

This was a diagnostic study using the cross-sectional method to determine the relationship between the SANS and PANSS instruments on the negative symptom subscale. The location was at the Inpatient Unit of the Mental Hospital of North Sumatera Province. This was a referral mental hospital for North Sumatera Province with 400 beds, and the implementation was conducted within 4 months. Subsequently, the non-probability purposive sampling type was used in this study.

The inclusion criteria were schizophrenic patients hospitalized and diagnosed based on the International Classification of Disease and Related Health Problems 10th edition (ICD-10) criteria, aged 18–45 years. Additionally, this study had the following exclusion criteria: having a general medical condition that can affect the subject's psychotic condition and a history of other psychiatric disorders.

A sample size is suggested according to the following scale, 50 subjects (very poor), 100 subjects (poor), 200 subjects (enough), 300 subjects (good), 500 subjects (very good), and 1,000 subjects or more (excellent) (11). A large sample was better than a small one, and it was recommended that many sample sizes be used (11). The minimum sample size needed was 400 subjects, and a scale with a sample size of 400 is considered good.

This study began with study preparation, including asking permission to translate and testing the validity and reliability of SANS to Nancy Andreas through electronic mail (e-mail). The next step was to obtain a research permit from the Head of the Department of Psychiatry, Faculty of Medicine, the Universitas Sumatera Utara before conducting the process of cross-cultural adaptation. This process aimed to achieve different language versions of the English instrument conceptually equivalent in each of the target countries/cultures. Therefore, the instrument should be natural and acceptable and practiced similarly. The focus was on cross-cultural and conceptual, not linguistic/literal equivalence. An excellent method to achieve this goal was to use forward and backward translations. The process used adaptations recommended by the Institute for Work and Health (IWH) as follows: (12, 13) Stage I was translation (forward translation). It required at least two translators to translate the instrument from the original to the target language. Both translators were bilingual, whose target language was their mother tongue (the translator's first language). The first translator should be a health professional familiar with the terminology in the instrument to be translated. The second translator should not understand the concept and have no medical background.

Furthermore, Stage II was a synthesis of two translations involving a third person who acted as a mediator in discussing the differences to produce one translation (12). Stage III was backward translation, where results from previous translations were translated back into the original language. This was conducted to ensure the accuracy of the translated version. Like the first stage, two bilingual translators were required, with the original language being their mother tongue. They also should not understand the concept or had a medical background (12). This stage was also conducted by two translators who are British citizens with an English language expert certificate. They were domiciled in Medan, North Sumatera, as English teachers in a private course institution in Medan, Indonesia. Finally, Stage IV was performed by requesting the expert committee review to obtain a cross-cultural translation of the instrument. The objective was to identify non-conforming translation concepts and any discrepancies between forward translation and a previously existing version of a measurement scale. The expert committee may question some words or phrases and suggest alternative translations. Experts should be provided with any material according to previous translations. The number of experts on the panel may vary but should include original translators and health professionals with experience in instrument development and translation (12, 13). At this stage, three expert committees, including EE, MA, and VC, were involved in checking the translation results. The results were collected and suggested improvements were consulted with B.L. to obtain the final result of the SANS translation in Indonesian.

This study was conducted using the final result of the Indonesian version of the SANS translation. After receiving detailed explanations, subjects who met the inclusion criteria filled out a written informed consent form. When it was impossible to fill out the consent form, the consent of the subject's family was obtained. Then, the diagnosis of schizophrenia was made using the ICD-10 diagnostic criteria. Next, the exclusion of other psychiatric disorders was assessed using the Mini International Neuropsychiatric Interview Version ICD-10 (MINI ICD-10). Before the examination, the assessor was trained or equalized the perception of items on the measurement scale and then examined with SANS and the Indonesian version of the negative scale PANSS with the help of a psychiatrist assessor at Prof. Dr. M. Ildrem Psychiatric Hospital Medan, North Sumatera. The negative scale SANS and PANSS questionnaire data were obtained before analyzing and processing.

Data were collected, tabulated, and statistically processed. The validity test was assessed by qualitative assessment of each instrument item by three experts in psychiatry. In the concurrent validity test, the Pearson correlation test was performed when the data distribution was normal; otherwise, the Spearman correlation test was performed to find the correlation coefficient between the numerical variables (SANS) and the negative symptom subscale of PANSS. The reliability test was measured by the internal consistency reliability of each statement item by calculating Cronbach's alpha and inter-rater reliability by the intra-class correlation coefficient (ICC). This study used the *Statistical Package for Social Science* (SPSS) program to process the data. Additionally, it received approval from the Research Ethics Committee at the University of North Sumatera with letter number 209/KEP/USU/2021.

## 3. Results

Demographic characteristics analyzed were age, gender, last education, employment status, marital status, ethnicity, Body Mass Index (BMI), duration of schizophrenia, and the onset of illness. The age, BMI, duration of illness, and disease onset were numerical scale variables. The Kolmogorov–Smirnov analysis was the normality test because there were more than 50 subjects. The mean and standard deviation presented when the data was normally distributed; otherwise, it presented in median and percentile. Gender, latest education, employment status, marital status, and ethnicity were categorical variables presented in a frequency distribution.

Table 1 showed the demographic characteristics of the subjects. Age was presented in the median value (minimum-maximum) because the data were not normally distributed, using the Kolmogorov–Smirnov test, with a median of 37 years (18–45). Most of the subjects, 270 samples (67.5%), were males. The highest education level was at the high school level with 156 samples (37%), and the highest employment status was the non-working group with 364 samples (91%). Furthermore, the marital status of 247 samples (61.8%) was unmarried, while the most predominant ethnic group was Batak with 261 samples (65.2%). The median value of BMI is 22.00, with a minimum and maximum value of 12.10 and 29.60, respectively. The duration of illness had a median of 9 years with a minimum and maximum value of 2 and 20 years, respectively. At the onset of illness, the median was 27 years with a minimum and maximum of 15 and 37 years, respectively.

**Table 1:** Demographic characteristics.

**Variable**	**People with schizophrenia** **(*n* = 400)**
Age (median)	37,00 (18,00–45,00)
**Gender**	
Male	270 (67.5%)
Female	130 (32.5%)
**Education**	
Elementary school	109 (27.2%)
Junior high school	79 (19.8%)
Senior high school	156 (39%)
Bachelor	56 (14%)
**Job status**	
Working	36 (9%)
Not work	364 (91%)
**Marital status**	
Married	153 (38.2%)
Single	247 (61.8%)
**Ethnic group**	
Batak	261 (65.2%)
Java	74 (18.5%)
Minang	20 (5%)
Malay	18 (4.5%)
Chinese	27 (6.8%)
BMI (median)	22.00 (12.10–29.60)
Duration of illness (median)	9.00 (2.00–20.00)
Disorder onset (median)	27.00 (15.00–37.00)

Categorical variables are represented by n (%), normally distributed numerical variables are represented by the mean ± standard deviation, non-normally distributed numerical variables are represented by the median value (minimum value-maximum value).

Content validity was accessed using a quantitative approach through an assessment of the measuring instrument conducted by several experts called the panel, who understand and explore the construct of the instrument assessed. Three experts conducted the validity of the SANS instrument and assessed each item using four value scales. This included a value of 4 when the item was very relevant, a value of 3 when the item was relevant, a value of 2 that was somewhat relevant but should be improved, and a value of 1 when the item was not relevant. The conformity assessment can be repeated (14).

*Content Validity Ratio* (CVR) was used to measure the agreement between raters/experts on the importance of certain instrument items. Content Validity Ratio (CVR) was calculated by the formula (*n*_e_ – *N*/2)/(*N*/2), where *n*_e_ was the number of experts who answered “quite relevant” and “very relevant,” namely scores 3 and 4, and *N* was the number of experts. I-CVI is the number of experts rated “fairly relevant” and “very relevant,” divided by the number of experts who participated in the assessment. The I-CVI score for good content validity was at least 0.78 for six experts or more and a score of 1.00 for three to five experts (14).

Table 2 showed the I-CVI value is 1.00 for each question item, and the value of S-CVI/UA obtained was 1.00, which indicated the instrument's content validity was good. The results of the CVR of each item were good, namely 1.00, and the value of S-CVI /Ave = (1.00 + 1.00 + 1.00)/3 = 1.00 (14).

**Table 2:** Assessment of the agreement on the validity of the contents of the Indonesian version of the SANS instrument.

**Item**	**Expert 1**	**Expert 2**	**Expert 3**	**Number in agreement**	**ItemI-CVI**	**CVR**
1	4	4	4	3	3/3 = 1.00	1.00
2	4	4	4	3	3/3 = 1.00	1.00
3	4	4	4	3	3/3 = 1.00	1.00
4	4	4	4	3	3/3 = 1.00	1.00
5	3	3	3	3	3/3 = 1.00	1.00
6	4	4	4	3	3/3 = 1.00	1.00
7	3	4	3	3	3/3 = 1.00	1.00
8	3	3	4	3	3/3 = 1.00	1.00
9	3	3	3	3	3/3 = 1.00	1.00
10	4	3	3	3	3/3 = 1.00	1.00
11	3	4	3	3	3/3 = 1.00	1.00
12	4	4	4	3	3/3 = 1.00	1.00
13	3	3	3	3	3/3 = 1.00	1.00
14	4	4	4	3	3/3 = 1.00	1.00
15	4	3	4	3	3/3 = 1.00	1.00
16	3	4	3	3	3/3 = 1.00	1.00
17	4	4	4	3	3/3 = 1.00	1.00
18	4	4	4	3	3/3 = 1.00	1.00
19	4	4	4	3	3/3 = 1.00	1.00
20	4	4	4	3	3/3 = 1.00	1.00
21	4	4	4	3	3/3 = 1.00	1.00
22	4	4	4	3	3/3 = 1.00	1.00
23	4	4	4	3	3/3 = 1.00	1.00
24	4	3	4	3	3/3 = 1.00	1.00
25	4	4	4	3	3/3 = 1.00	1.00
∑	25	25	25			
Proportion relevant	1	1	1	Mean I-CVI = 1.00		
*Mean expert proportion = 1.00*						
S-CVI/UA (scale-level content validity index, universal agreement calculation method) = 25/25 = 1.00						
S-CVI/Ave = (1.00 + 1.00 + 1.00)/3 = 1.00						

The concurrent validity test compared the Indonesian version of the scale for assessing negative symptoms (SANS) scores with a standard gold instrument. This study used the Indonesian PANSS negative symptom subscale (PANSS-NS) instrument. The normality test used was the Kolmogorov–Smirnov test because the number of participants was more than 50, and both results were *p* < 0.001. First, it was concluded that the data distribution was not normal, then the log_10_ function continued to normalize the data distribution and obtain a fixed result of *p* < 0.001. However, the data distribution was still abnormal with the assumption of linearity based on the scatter graph (spread). Therefore, the relationship between linear variables was obtained before conducting the Spearman correlation test.

Table 3 obtained a *p*-value < 0.001, indicating that the correlation between the SANS and PANSS-NS scores was significant. Furthermore, the Spearman correlation value of 0.763 indicated a strong positive correlation.

**Table 3:** Concurrent validity with Spearman correlation between SANS and PANSS-NS.

**Variable**	**Subjects (*n* = 400)**	***r* value**	***p*-Value**
SANS	11.00 (2.00–92.00)	0.763	<0.001
PANSS-NS	9.50 (7.00–37.00)		

Table 4 showed the internal consistency reliability of the Indonesian version of the SANS instrument and the Chronbach alpha of 0.969, which was ideal as it is >0.7. Table 5 illustrated that all questions have a corrected item-total item correlation more significant than the minimum correlation coefficient, which was considered valid (0.3). Subsequently, 25 questions were valid and had reliability of 0.969 (Cronbach alpha).

**Table 4:** Reliability statistics.

**Cronbach's alpha**	**No. of items**
0.969	25

**Table 5:** Internal consistency of the Indonesian version of SANS.

	**Scale mean if item deleted**	**Scale variance if item deleted**	**Corrected item-total correlation**	**Cronbach's alpha if item deleted**
Q1	16.06	384.595	0.598	0.969
Q2	17.10	374.018	0.711	0.968
Q3	16.67	378.573	0.602	0.969
Q4	16.80	381.943	0.528	0.970
Q5	17.25	371.276	0.769	0.968
Q6	16.95	379.837	0.578	0.969
Q7	17.16	369.283	0.804	0.967
Q8	16.91	372.536	0.823	0.967
Q9	17.15	367.373	0.840	0.967
Q10	17.23	373.302	0.806	0.967
Q11	17.32	371.464	0.805	0.967
Q12	17.25	366.061	0.860	0.967
Q13	17.22	367.503	0.875	0.967
Q14	17.58	377.047	0.876	0.967
Q15	17.45	381.602	0.711	0.968
Q16	17.34	369.492	0.850	0.967
Q17	17.47	373.874	0.877	0.967
Q18	17.40	378.275	0.742	0.968
Q19	16.81	372.687	0.673	0.969
Q20	17.01	377.677	0.637	0.969
Q21	17.35	377.507	0.721	0.968
Q22	17.36	375.824	0.883	0.967
Q23	17.45	374.504	0.839	0.967
Q24	16.64	390.457	0.352	0.971
Q25	17.31	377.298	0.811	0.967

The reliability of the SANS instrument was measured using an inter-rater reliability design. This approach was preferred over test-retest because the assessment depended on observations of patient behavior compared to self-reports and subjective complaints. Primarily, it determined how well two or more independent observers or raters will agree on some aspect of the patient's behavior. Furthermore, the test-retest design provides an additional source of variance, such as changes in patient behavior over time, giving rise to an undesirable source of variance. This did not allow a net assessment of variance between rater observations. Furthermore, the inter-rater reliability design was preferred because assessing the observed variance was the main objective (7).

Inter-rater (equivalence) measurement test on the SANS instrument used the ICC. In examining the reliability, the rule of thumb should involve a minimum of 30 samples assessed by a minimum of 3 raters (15). A total of 50 samples and 3 raters were assigned, and each instrument item on the inter-rater was measured using the ICC. This was achieved with a two-way mixed-effects model, single rater type, and absolute agreement provisions (15, 16).

Table 6 showed that the ICC of all items obtained inter-rater reliability of 0.985. When the 95% confidence interval (CI 95%) of the ICC estimate was in the range of 0.974–0.991, then the reliability could be considered “very good.” The global assessment of each domain found that if the highest ICC was in the “flattening and blunting of effect” domain with a value of 0.881 and a CI 95% in the range of 0.819–0.926, then reliability could be considered “good” to “very good.” Meanwhile, the lowest domain was in the “anhedonia-asocial” domain with a value of 0.830 and CI 95% in the range of 0.746–0.893; therefore, reliability could be considered “good.” The highest ICC was found in “Indifference during mental status examination” domain with a value of 0.970 and CI 95% in the range of 0.952–0.982 to consider the reliability “very good.” The lowest item was “Indifference at work or school” domain with a score of 0.701 and a CI 95% in the range of 0.545–0.814; therefore, the reliability could be considered “sufficient” to “good” (15, 16).

**Table 6:** Inter-rater reliability of Indonesian version of SANS.

**No**.	**Item**	**Intra-class correlation**	**95% CI**
	**Flattening and blunting of effect**		
Q1	Stable facial expressions	0.890	0.832–0.932
Q2	Reduced spontaneous movement	0.893	0.781–0.944
Q3	Rarely expressive behavior	0.767	0.597–0.867
Q4	Poor eye contact	0.862	0.790–0.914
Q5	Inability to respond to affective	0.906	0.853–0.942
Q6	Inappropriate affect	0.910	0.862–0.945
Q7	Decreased intonation	0.847	0.767–0.905
Q8	Global affect flattening assessment	0.881	0.819–0.926
	**Alogia**		
Q9	Low conversation	0.821	0.598–0.912
Q10	Low conversation content	0.828	0.741–0.892
Q11	Blocking of thought	0.852	0.743–0.915
Q12	Improved latent response	0.883	0.765–0.938
Q13	Global alogia assessment	0.837	0.621–0.921
	**Avolition—apathy**		
Q14	Care and hygiene	0.794	0.696–0.869
Q15	Diligence at work or school	0.701	0.545–0.814
Q16	Lack of physical energy (physical anergia)	0.878	0.768–0.934
Q17	Global avolition—apathy assessment	0.857	0.783–0.911
	**Anhedonia—asocial**		
Q18	Recreational interests and activities	0.821	0.733–0.887
Q19	Sexual activity	0.851	0.775–0.907
Q20	The ability to feel intimacy and closeness	0.764	0.645–0.842
Q21	Relationships with friends and neighbors	0.898	0.844–0.937
Q22	Global anhedonia—asocial assessment	0.830	0.746–0.893
	**Concern**		
Q23	Social indifference	0.870	0.755–0.930
Q24	Indifference during mental status examination	0.970	0.952–0.982
Q25	Global care assessment	0.876	0.804–0.924
	Combined score	0.985	0.974–0.991

The receiver operating characteristic (ROC) and (AUC) were inseparable methods. The ROC method results from a trade-off between the sensitivity and specificity values at various alternative intersection points presented in the graph. The AUC was the result of the region generated from the ROC curve.

**Figure 1:**
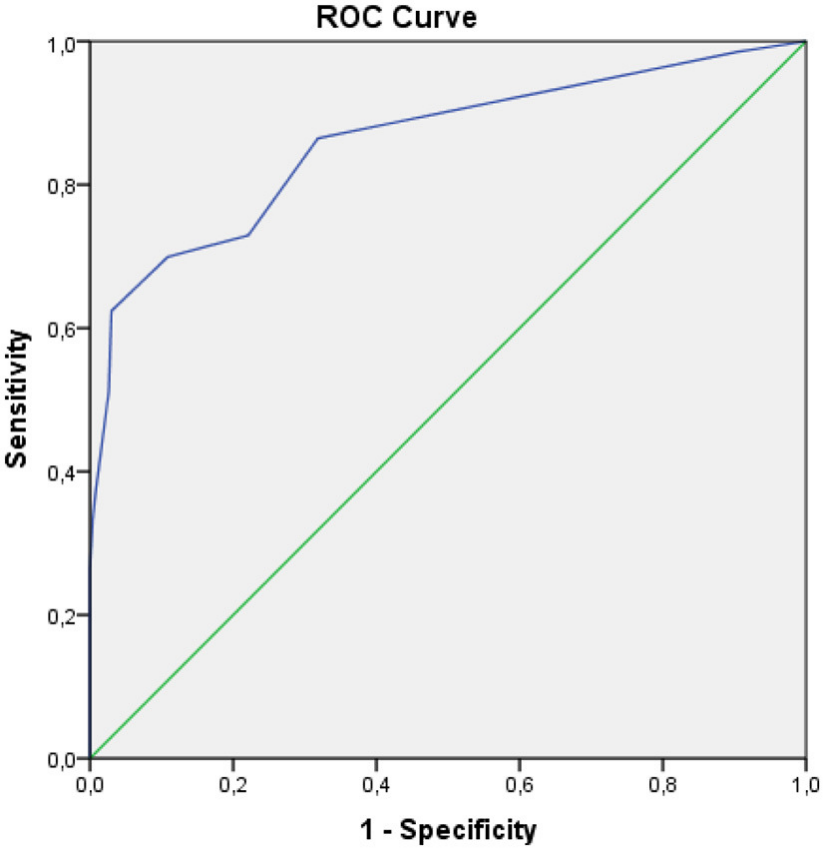
ROC curve.

In Figure 1, SANS had a good diagnostic value because the curve was far from the 50% line and was in the 100% line. The AUC value obtained from the ROC method was 86.0% (95% 81.7%−90.2%), *p* < 0.001. Furthermore, the hypothesis test compared the AUC obtained by the index to the AUC of 50%, with a *p*-value of <0.05, which indicated that the AUC value of the SANS instrument was significantly different from that of 50%. However, the AUC value was satisfactory because it was more significant than the expected minimum value.

**Figure 2:**
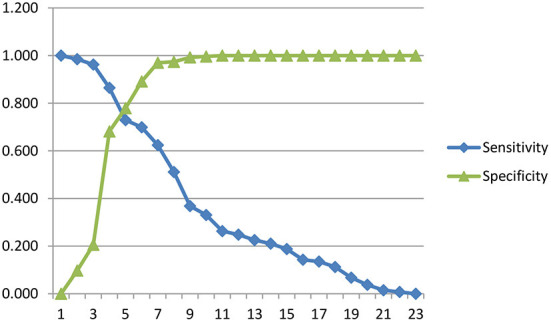
Sensitivity and specificity curve.

Figure 2 presented a sensitivity and specificity curve. The optimal intersection point was the value at which the sensitivity and specificity curves intersect. This could be determined by drawing a vertical line from the intersection point between points 5 and 6.

Table 7 presented the optimal cut-off point between 5 and 6, where the value of 5 was ≥10.5 with sensitivity and specificity of 72.9 and 77.9%, respectively. The value of point 6 was ≥11.5 with 69.9 and 89.1% sensitivity and specificity, respectively. Therefore, it was concluded that the cut-off point that showed negative symptoms had a total SANS score of 10.5.

**Table 7:** The sensitivity and specificity values of various alternative intersection points.

**No**	**Variable**	**Cut-off**	**Sensitivity**	**Specificity**	**AUC**	**95% CI**
1		6.00	1.000	0.000		
2		7.50	0.985	0.097		
3		8.50	0.962	0.206		
4		9.50	0.865	0.682		
5	SANS	10.50	0.729	0.779	86.0	81.7–90.2
6		11.50	0.699	0.891		
7		12.50	0.624	0.970		
8		13.50	0.511	0.974		
9		14.50	0.368	0.993		
10		15.50	0.331	0.996		
11		16.50	0.263	1.000		
12		21.00	0.248	1.000		
13		25.50	0.226	1.000		
14		26.50	0.211	1.000		
15		27.50	0.188	1.000		
16		28.50	0.143	1.000		
17		29.50	0.135	1.000		
18		30.50	0.113	1.000		
19		31.50	0.068	1.000		
20		32.50	0.038	1.000		
21		33.50	0.015	1.000		
22		35.50	0.008	1.000		
23		38.00	0.000	1.000		

The AUC score of 86.0% was good, meaning that when a negative symptom score was used to assess the symptoms in 100 subjects, the examination will give the correct conclusion for 86 people.

## 4. Discussion

Obtaining a valid and accurate Indonesian version of the SANS instrument is crucial for objective assessment of schizophrenia symptoms. As mentioned before, the purpose of this study was to establish the SANS instrument's content validity, concurrent, internal consistency reliability, inter-rater, cut-off value, sensitivity and specificity. The subjects were people with schizophrenia aged 18–45 years to minimize bias due to secondary negative symptoms caused by non-schizophrenic diseases or a degenerative process (17). Demographic characteristics showed that most subjects were male (67.5%), and in terms of age, the median was 37 years with a minimum and maximum of 18 years and 45 years, respectively. The study by Charlson et al. with the title Global Epidemiology and Burden of Schizophrenia reported data on the prevalence of schizophrenia globally. The global prevalence was ~70.8% for 25–54 years, highest in the 40s and decreasing in the older age group (2). The median duration of illness was 9 years with a minimum and maximum of 2 and 20 years, respectively. Moreover, the median disease onset was 27.00 years with a minimum and maximum of 15.00 and 37.00 years, respectively. There was no specific age-associated with schizophrenia because this condition can occur at any age. Almost all schizophrenia disorders begin with a prodromal stage and impact the social conditions of each sufferer. The onset is mainly at 15–25 years in males and 15–30 years in females (2).

Three experts conducted the content validity test to find the values of I-CVI, S-CVI, CVR, S-CVI/UA, and S-CVI/Ave. Table 2 explained that the I-CVI and CVR values obtained are 1.00 for each question item with a mean I-CVI value of 1.00. Under the I-CVI requirements of the content validity test, using less than five experts can be considered “good” or “very relevant” when the I-CVI value is 1.00. The S-CVI/Ave obtained was 1.00, indicating that the content validity of this instrument is good because the minimum value of S-CVI/Ave is 0.90. Therefore, the content validity test shows that the questions on the Indonesian version of the SANS instrument have relevance to the construction of negative symptoms in people with schizophrenia (14). The study conducted by Charernboon validated SANS in Thai obtained content validity results with a mean I-CVI value of 0.94. This result is consistent with this study, which has a mean I-CVI of 1.00, where it is concluded that both have good content validity (10).

The concurrent validity test used the Spearman correlation test to determine the correlation between the Indonesian version of the SANS and the standard gold instrument. This study used the negative symptom subscale PANSS instrument, and the SPSS calculation obtained an *r* value = 0.76 with a *p*-value of <0.001, as shown in Table 3. It indicated a strong significant correlation between the SANS and PANSS scores on the negative symptom subscale with a positive correlation between the two instruments. Therefore, when the Indonesian version of the SANS score gets high results, the negative symptom subscale PANSS score will also give high results. However, there are varying results from previous studies regarding this validity value. In 1991, Philips et al. measured the validity and reliability of the Chinese version of the SAPS and SANS. They obtained a significant correlation between the SANS score and the Brief Psychiatric Rating Scale (BPRS) score (*p* < 0.001) at admission and discharge from the hospital, with positive correlation results and weak to moderate correlation strength (*r* = 0.17–0.46).

Furthermore, Thammanard in Thailand found a significant correlation between SANS scores and the Addenbrooke's Cognitive Examination (ACE; *p* = 0.002) with a negative correlation and moderate correlation strength (*r* = −0.48). The difference in the results obtained is possible because of an ethnocentric bias, differences in the gold standard instruments used, and the tests conducted (7, 8, 10). Philips et al. described this ethnocentric bias as items in the initial version of the SANS instrument written using Western concepts. Subsequently, the selection of items for the final version was based on the studies using subjects from Western countries; hence, using this instrument in the group or other ethnicities who are not English speakers is often problematic, and their validity is questionable. Ideally, health status instruments should be developed independently in each culture and then cross-culturally. However, studies in developing countries rarely have the resources needed. The urgent need for clinical and sociocultural measures in non-Western cultures has led to the widespread use of Western translation instruments. However, careful back-translation of a reliable and valid instrument in the West does not result in a reliable and valid instrument in non-Western cultures following the variation in this study and several others. The clinical usefulness of a translated instrument depends on the scientific rigor evaluated and revised in the target culture (8).

Cronbach's alpha value for the Indonesian version of SANS was 0.969, with the interpretation that the internal consistency value of this instrument is very high. However, Cronbach's alpha coefficient is often used to assess internal consistency, even though there is no common agreement regarding its interpretation. For example, several studies have determined that Cronbach's alpha of >0.7 is ideal, but a value close to 0.6 is satisfactory (18). Classification of Cronbach's alpha comprises very low (≤0.3), low (>0.3–≤0.6), moderate (>0.6–0.75), high (>0.75–≤0.9), and very high (>0.9) (19). The high internal consistency in the Indonesian version of SANS is in line with the findings of previous studies conducted by Andreasen, the creator of this instrument, and obtained a high internal consistency score of the overall SANS items (Cronbach's alpha = 0.885). Additionally, Philips et al. measured the validity and reliability of the Chinese version of the SAPS and SANS, where the overall score of the Chinese version of the SANS had a very high internal consistency (Cronbach's alpha = 0.96). Additionally, the SANS validation study in Thailand conducted by Thammanard obtained a very high overall internal consistency score of items (Cronbach's alpha = 0.95). This supports the suggestion of several previous studies that the negative symptom construct is more homogeneous than the positive one (7, 8, 10). The corrected item-total correlation value of the Indonesian version of SANS is above the minimum correlation coefficient considered valid, which is 0.3. Therefore, 25 assessment items on the SANS instrument are deemed valid.

In Table 6, the intraclass correlation coefficient (ICC) of all items shows inter-rater reliability of 0.985. If the 95% confidence interval (CI 95%) of the ICC estimate is in the range of 0.974–0.991, then the reliability level can be considered “very good.” In the global assessment in each domain and per item, the SANS instrument has a fairly good to a very good level (ICC values between 0.545–0.970). This study is in line with Andreasen, who obtained an overall ICC value of 0.92 (very good) and a value per item and per domain, which had a good to a very good level of reliability (ICC values between 0.701–0.926). Meanwhile, Philips et al. obtained a very good ICC value of the overall items (ICC = 0.93) and a value per item and per domain that had a good to very good reliability (ICC values between 0.85–0.95). In 1996, Norman et al. compared the inter-rater reliability of SAPS, SANS, and PANSS and reported slightly different results, namely, the ICC value of the whole item was 0.68 (medium), and the value per item and domain had the reliability of bad to good (ICC value between 0.28–0.74).

The AUC value obtained from the ROC method was 86.0% (95% 81.7%−90.2%), *p* < 0.001. Test the hypothesis to compare the AUC obtained by the index to the AUC value of 50%, with a *p*-value of <0.05, which indicates that the AUC value of the SANS score is significantly different from that of 50%. Interpretation of AUC with a statistical approach concluded that it has a good power of diagnostic value (>80%−90%). The size of the AUC area will show the validity of the conclusions given by a diagnostic instrument for clinical purposes. The AUC of 86.0% is a good value, meaning that when a negative symptom score is used to diagnose the negative symptoms from 100 subjects, then the right conclusion can be obtained about 86 people. Sensitivity is the ability of an instrument to show positive results in a genuinely positive population, while specificity is the ability of an instrument to show negative results in a genuinely negative population (20).

In Figure 2, the optimal cut-off point is between 5 and 6. In point 5, the value was ≥10.5, with sensitivity and specificity values of 72.9 and 77.9%, respectively. Meanwhile, point 6 was ≥11.5 with 69.9 and 89.1% sensitivity and specificity values, respectively. There is a consideration to prioritize a higher sensitivity value than the specificity value. Therefore, it can be concluded that the cut-off point that shows the negative symptoms is a total SANS score of ≥10.5, with a sensitivity of 72.9% and a specificity of 77.9%. Sensitivity of 72.9% signifies that in 100 people with negative symptoms, SANS gives correct results in 73 people. In comparison, the specificity of 77.9% means that SANS gives correct results in 100 people without negative symptoms in 78 people.

This study has implemented the content and concurrent validity test, internal consistency, and inter-rater reliability test. Additionally, this study obtained good AUC values with the ROC method, cut-off values, and sensitivity and specificity values of the Indonesian version of the SANS instrument. Contrarily, the limitation of this study was that it used non-probability sampling method and was only conducted in one particular area or place due to the current pandemic conditions.

## 5. Conclusion

This study showed that the content validity test was good (mean I-CVI = 1.00), and the concurrent validity test that compared the SANS and PANSS instruments on the negative symptom subscale obtained significant results (*p* < 0.001), with a strong correlation (*r* = 0.763). Furthermore, the reliability test for internal consistency was very high (Cronbach alpha = 0.969), the overall item inter-rater reliability test was “very good” (ICC = 0.985), and the cut-off value was 10.5 with a sensitivity and specificity of 72.9 and 77.9%, respectively. In conclusion, the Indonesian version of the SANS instrument is valid and reliable for measuring negative symptoms in people with schizophrenia.

## Data availability statement

The datasets presented in this study can be found in online repositories. The names of the repository/repositories and accession number(s) can be found in the article/supplementary material.

## Ethics statement

The studies involving human participants were reviewed and approved by Study Ethics Committee at the Universitas Sumatera Utara. The patients/participants provided their written informed consent to participate in this study.

## Author contributions

MA, EE, VC, and MS were involved and contributed equally to the study. RH and AK conducted forward translation. All authors contributed to the article and approved the submitted version.

## Dedication

We would like to express our deep condolences to our beloved teacher Bahagia Loebis that passed away after the study finished.

## References

[1] Robertson GS Hori SE Powell KJ. Schizophrenia: an integrative approach to modelling a complex disorder. J Psychiatry Neurosci. (2006) 31:157–67. 16699601 PMC1449879

[2] Chong HY Teoh SL Wu DB-C Kotirum S Chiou C-F Chaiyakunapruk N. Global economic burden of schizophrenia: a systematic review. Neuropsychiatr Dis Treat. (2016) 12:357–73. 10.2147/NDT.S96649 26937191 PMC4762470

[3] Kementerian Kesehatan Republik Indonesia. Hasil Utama RISKESDAS 2018. Jakarta (2018).

[4] Kumari S Malik M Florival C Manalai P Sonje S. An assessment of five (PANSS, SAPS, SANS, NSA-16, CGI-SCH) commonly used symptoms rating scales in schizophrenia and comparison to newer scales (CAINS, BNSS). J Addict Res Ther. (2017) 08:324. 10.4172/2155-6105.1000324 29430333 PMC5805140

[5] Dollfus S Lyne J. Negative symptoms: history of the concept and their position in diagnosis of schizophrenia. Schizophr Res. (2017) 186:3–7. 10.1016/j.schres.2016.06.024 27401529

[6] Galderisi S Färden A Kaiser S. Dissecting negative symptoms of schizophrenia: history, assessment, pathophysiological mechanisms and treatment. Schizophr Res. (2017) 186:1–2. 10.1016/j.schres.2016.04.046 27185482

[7] Andreasen NC. Negative symptoms in schizophrenia. Definition and reliability. Arch Gen Psychiatry. (1982) 39:784–8. 10.1001/archpsyc.1982.04290070020005 7165477

[8] Phillips MR Xiong W Wang RW Gao YH Wang XQ Zhang NP. Reliability and validity of the Chinese versions of the Scales for Assessment of Positive and Negative Symptoms. Acta Psychiatr Scand. (1991) 84:364–70. 10.1111/j.1600-0447.1991.tb03161.x 1746289

[9] Norman RMG Malla AK Cortese L Diaz F. A study of the interrelationship between and comparative interrater reliability of the SAPS, SANS and PANSS. Schizophr Res. (1996) 19:73–85. 10.1016/0920-9964(95)00055-0 9147498

[10] Charernboon T. Preliminary study of the Thai version of the Scale for the Assessment of Negative Symptoms (SANS-Thai). Glob J Health Sci. (2019) 11:19. 10.5539/gjhs.v11n6p19

[11] Tsang S Royse CF Terkawi AS. Guidelines for developing, translating, and validating a questionnaire in perioperative and pain medicine. Saudi J Anaesth. (2017) 11:S80–9. 10.4103/sja.SJA_203_17 28616007 PMC5463570

[12] Beaton D Bombardier C Guillemin F Ferraz MB. Recommendations for the cross-cultural adptation of teh DASH & QuickDASH outcome measures. Institute for Work and Health. (2007). Available at: https://dash.iwh.on.ca/sites/dash/files/downloads/cross_cultural_adaptation_2007.pdf

[13] World Health Organization. Management of Substance Abuse Process of Translation and Adaptation of Instruments. (2013) Available at: https://www.mhinnovation.net/sites/default/files/files/WHO%20Guidelines%20on%20Translation%20and%20Adaptation%20of%20Instruments.docx

[14] Polit DF Beck CT. The content validity index: are you sure you know what's being reported? Critique and recommendations. Res Nurs Health. (2006) 29:489–97. 10.1002/nur.20147 16977646

[15] Perinetti G. StaTips Part IV: selection, interpretation and reporting of the intraclass correlation coefficient. South Eur J Orthod Dentofac Res. (2018) 5:3–5. 10.5937/sejodr5-17434

[16] Koo TK Li MY. A guideline of selecting and reporting intraclass correlation coefficients for reliability research. J Chiropr Med. (2016) 15:155–63. 10.1016/j.jcm.2016.02.012 27330520 PMC4913118

[17] Correll CU Schooler NR. Negative symptoms in schizophrenia: a review and clinical guide for recognition, assessment, and treatment. Neuropsychiatr Dis Treat. (2020) 16:519–34. 10.2147/NDT.S225643 32110026 PMC7041437

[18] de Souza AC Alexandre NMC de Brito Guirardello E. Psychometric properties in instruments evaluation of reliability and validity. Epidemiol Serv Saude. (2017) 26:649–59. 10.5123/S1679-49742017000300022 28977189

[19] Gottems LBD Carvalho EMP Guilhem D Pires MRGM. Good practices in normal childbirth: reliability analysis of an instrument by Cronbach's Alpha. Rev Lat Am Enfermagem. (2018) 26:e3000. 10.1590/1518-8345.2234.3000 29791667 PMC5969829

[20] Dahlan S. Metode Multi-Aksial Sopiyudin Dahlan: Pintu Gerbang Memahami Epidemiologi, Statistik, Dan Metodologi (2014).

